# Effects of polylactic acid scaffolds with various orientations and diameters on osteogenesis and angiogenesis

**DOI:** 10.3389/fbioe.2024.1495810

**Published:** 2025-01-06

**Authors:** Yun Rong Xu, Dai Yuan Tang, Zhen Ping Xiao, Zai Tian Huang, Heng Rui Zhang, Zi Wen Tang, Fei He

**Affiliations:** ^1^ Qujing Affiliated Hospital of Kunming Medical University, Qujing, China; ^2^ The First Affiliated Hospital of Kunming Medical University, Kunming, Yunnan, China

**Keywords:** micromorphology, polylactic acid, orientation, diameter, osteogenesis, vascular generation

## Abstract

Researchers in the field of regenerative medicine have consistently focused on the biomimetic design of engineered bone materials on the basis of the microstructure of natural bone tissue. Additionally, the effects of the micromorphological characteristics of these materials on angiogenesis have garnered increasing attention. *In vitro*, the orientation and diameter of scaffold materials can exert different effects on osteogenesis and vascularisation. However, more comprehensive investigations, including *in vivo* studies, are required to confirm the results observed *in vitro*. Accordingly, in the present study, fibre scaffolds with various orientations and diameters were prepared by electrospinning with polylactic acid. The effects of the micromorphological characteristics of these scaffolds with different orientations and diameters on osteogenesis and vascularisation were systematically studied via *in vivo* experiments. The scaffolds with aligned micromorphological features positively affected osteogenesis and vascularisation, which indicated that such characteristics could be considered crucial factors when designing materials for bone repair.

## 1 Introduction

The skeletal system plays various roles in the human body, whereas critical bone defects caused by pathologies, including trauma, infection, and tumours are a formidable challenge in clinical therapeutics (Wang et al., 2024). Autologous bone transplantation is the gold standard for treatment; however, its widespread application is restricted owing to the scarcity of sources, and bone allografts may face several issues, including the potential for immune rejection (Zhang et al., 2023). Advancements observed over the past few decades in bone tissue engineering can address this issue. Many promising prospects for bone regeneration have been developed by integrating various osteogenic strategies with combinations of materials, bioactive components, and cells. Among these, biomimetic studies focusing on natural bone tissue are crucial because the physical cues provided by the materials that mimic natural tissue are essential components in bone tissue reconstruction, and morphologies and structures matching those of the bone tissue facilitate bone restoration and regeneration (Fernandez-Yague et al., 2015). For example, the microstructure of the material, the stiffness of the substrate, and the topography affect cellular development during tissue regeneration (Ding et al., 2020). Additionally, cellular alignment can be controlled by the microstructure of the material, and nanostructures affect cell adhesion and differentiation (Sankar et al., 2018). The stiffness of the substrate is a critical determinant in regulating cellular osteogenic differentiation (Hsieh et al., 2016; Liu et al., 2018; Yang et al., 2020), and appropriate pore size and porosity can modulate osteoblast aggregation to promote osteogenic effects (Ratheesh et al., 2021). Furthermore, materials with unique surface morphological configurations can provide favourable biophysical cues, and by interacting with the microenvironment to regulate cellular behaviour, tissue regeneration can be induced in the absence of modifications or external stimuli (Kim et al., 2015).

The bone tissue comprises an intricate arrangement of highly ordered mineralised collagen fibres and exhibits mechanical properties determined by the hierarchical anisotropic structure, which is instrumental in directing cellular differentiation and nutrient transport; thus, it is a focal point of current research (Liu et al., 2023). Zhang et al. reported that highly ordered TiO_2_ nanotube arrays prepared via the high-voltage anodisation method exhibited excellent osteogenic effects (Zhang et al., 2015). Similarly, biomimetic scaffolds with oriented microchannels, which are prepared by integrating three-dimensional (3D) printing with directional freezing, can upregulate the expression of osteogenesis-related genes and promote osteogenic differentiation both *in vitro* and *in vivo* (Cheng et al., 2024). Currently, various nanoscale and microscale materials have been extensively used to determine the effects of topography on osteogenic differentiation (Gao et al., 2023). Using scaffolds composed of fibres with consistent orientation, cellular proliferation can be improved, and alkaline phosphatase activity can be enhanced (Guo et al., 2015). Xie et al. (2021a) investigated the effects of nanoscale (0.6 μm) and microscale (1.2 μm) scaffolds aligned against random polylactic acid electrospun membrane scaffolds on the osteogenic differentiation of bone marrow mesenchymal stem cells and reported that compared with random nanoscale and microscale scaffolds and aligned microscale scaffolds, aligned nanoscale scaffolds could remarkably improve osteogenic differentiation, which indicated that the oriented characteristics of the material surface topography play a crucial role in scaffold-based osteogenic differentiation.

Bone is a highly vascularised tissue, and osseous vessels play crucial roles in bone development, regeneration, and remodelling (Filipowska et al., 2017). Endothelial cells and vascular networks exhibit dynamic responses to external mechanical stimuli (Boerckel et al., 2011). Furthermore, the characteristics of materials within the surrounding extracellular matrix affect angiogenesis (Mammoto et al., 2009; Edgar et al., 2013). Additionally, substrate stiffness plays a critical role in regulating the vascular morphogenesis of human umbilical vein endothelial cells (HUVECs) (Du et al., 2016), whereas the adhesion and orientation of HUVECs are highly dependent on the orientation and morphology of fibre scaffolds (Montero et al., 2012). Moreover, endothelial cells can be reorganised on anisotropic scaffolds, leading to the formation of highly organised cellular structures (Gaharwar et al., 2014). However, studies on the *in vivo* effects of different orientations and diameters on angiogenesis are lacking.

Furthermore, electrospinning effectively simulates the characteristics of the extracellular matrix to prepare tissue engineering scaffolds with unique surface structures (Lyu et al., 2013). Owing to recent rapid developments in electrospinning technology and the utilisation of an electric field to draw, accelerate, and stretch polymer solutions, biomimetic scaffolds with large surface areas, complex surface topographies, and ease of functionalisation can be produced (Wang et al., 2021; Lin et al., 2020). Furthermore, precise control over the diameter and orientation of the fibres can be realised (Samavedi et al., 2014), and this method is widely applied in the fabrication of materials for bone tissue engineering (Anjum et al., 2022). Additionally, biocompatibility and biodegradability are crucial factors for developing tissue engineering scaffolds. Among various bone repair materials, the organic polymer polylactic acid has been extensively used in bone regeneration owing to its excellent biocompatibility (Lin et al., 2020). Additionally, it has been approved by the Food and Drug Administration (FDA) for biomedical applications (Ali et al., 2019).

Thus, in the present study, we aimed to use polylactic acid as the original material and employ electrospinning technology to produce nano and micro-nano scaffolds with varying orientations. The resulting surface topography was determined, followed by the implementation of these scaffolds in an animal bone defect model. The effects of various orientations and diameters of the polylactic acid scaffolds on osteogenesis and angiogenesis were determined via microcomputed tomography (CT) scanning, histological staining, and immunofluorescence staining.

## 2 Materials and methods

### 2.1 Materials

Biograde poly-L-lactic acid (PLLA, average molecular weight = 16 kDa) was purchased from Jinan Daigang Biomaterial Co. Ltd. and was applied without further purification. Hexafluoroisopropanol (HFIP) and phosphate-buffered saline (PBS) were purchased from Shanghai Mclean Biochemical Technology Co. Ltd. and VivaCell Biosciences, respectively. All other reagents were obtained from Wuhan Servicebio Co. Ltd. unless otherwise indicated.

### 2.2 Preparation and characterisation of the scaffolds

According to the electrospinning parameters established in a previous study (Chen et al., 2023), scaffolds with different orientations and diameters of micromorphology were fabricated. Briefly, PLLA was dissolved in HFIP to obtain a mixed solution with a concentration of 16% w/v, and after vortexing for 30 min, the solution was further homogenised using an ultrasonic oscillator for 1 h. Subsequently, the solution was placed into a syringe for electrospinning. Scaffolds were prepared by adjusting the voltage, syringe pump speed, syringe tip-to-collector distance, and collector rotation speed (Table 1). All the as-prepared electrospun scaffolds were maintained at room temperature for 6 days to remove residual organic solvents. The nanoscale and microscale scaffolds with concentrated orientations were named *Aligned 600 (A600)* and *Aligned 1,200 (A1200)*, respectively. Conversely, scaffolds with random orientation were named *Random 600 (R600)* and *Random 1,200 (R1200)*, respectively. The scaffolds were cut into 1 cm × 1 cm samples and sputter coated with gold, and their surface morphological characteristics, including the selection of random areas, were examined by scanning electron microscopy (SEM, ZEISS Sigma 300, Germany) at different magnifications. For each experimental group, 100 fibres were randomly selected, and their diameters and angular orientations were subsequently measured via ImageJ software (https://imagej.net/software/imagej/). Moreover, two-dimensional fast Fourier transform (2D FFT) analysis of the SEM images was performed to evaluate the degree of fibre orientation within the scaffolds (Ayres et al., 2008).

**TABLE 1:** Parameters for the preparation of polylactic acid electrospun fibre scaffolds.

Experimental group	Bore size	Voltage	Syringe pump speed	Syringe tip-to-collector distance	Syringe size	Collector rotation speed
A600	28G	6 kV	0.05 mm/min	14 cm	10 mL	2800r/min
R600	30G	9 kV	0.03 mm/min	20 cm	5 mL	100r/min
A1200	25G	7 kV	0.25 mm/min	12 cm	10 mL	2800r/min
R1200	28G	6 kV	0.05 mm/min	14 cm	10 mL	100r/min

### 2.3 Construction of a critical calvarial defect model in rats

All the animal experimental procedures were approved by the Animal Experiment Ethics Review Committee of Kunming Medical University (kmmu20221831). Sprague-Dawley (SD) rats (male, 8 weeks old with a body weight of about 210 g) were obtained from the Department of Laboratory Animal Science at Kunming Medical University and were maintained in an SPF facility at the institution. The critical calvarial defect model in rats was established following a standardised protocol (Liu et al., 2020). Forty-five SD rats were randomised into five groups. After anaesthesia was induced by injecting pentobarbital intraperitoneally, the hair on the cranial region of the head was shaved, and the area was cleaned and disinfected. A sagittal incision was made to gradually expose the skull, and symmetrical defects were created via a 5-mm diameter trephine. The scaffolds, which were previously subjected to ultraviolet irradiation, cleaned with 75% ethanol, rinsed and dried with PBS, were implanted as follows: A600, R600, A1200, and R1200. Additionally, a control group without scaffold implantation was established. The surgical wounds were subsequently closed layer by layer, and prophylactic penicillin was injected for 3 days postoperatively to prevent infection. At weeks 4, 8, and 12 postoperatively, three SD rats from each group were humanely euthanised, and their calvarial samples were collected and fixed in 10% polyformaldehyde for subsequent experiments.

### 2.4 Micro-CT analysis

Micro-CT (Skyscan 1,176 Micro-CT System, Skyscan, Bruker, Germany) was performed to scan the calvarial samples at distinct time intervals to evaluate bone growth, and the parameters for the scans were set as follows: a scanning voltage of 65 kV, a scanning current of 385 μA, an exposure time of 340 ms, and a resolution of 9 µm. The software provided with the instrument was used to reconstruct the 3D structure of the calvaria, and a cylindrical region of interest bounded by the edges of the defect was selected to calculate the bone-volume-to-tissue-volume ratio and the trabeculae number for visual analysis, which can accurately reflect the morphological characteristics of bone, deduce the parameters of bone growth and development level, and conduct research and analysis of new bone.

### 2.5 Histological and immunofluorescence analyses

After CT scanning, the calvarial samples were decalcified using 10% ethylenediaminetetraacetic acid, followed by paraffin embedding. Sections were cut along the coronal plane of the calvarial defect area using a microtome for hematoxylin and eosin (H&E) and Masson’s trichrome staining. Images were obtained via an upright optical microscope (Nikon Eclipse E100, Japan), and ImageJ software was used to quantitatively analyse the area of the newly formed bone tissue and the distance of the bone defect. Picrosirius red staining was performed to observe the growth trend of collagen fibres in the new bone tissue via a polarised light microscope (Nikon Eclipse ci, Japan), whereas TRAP staining was performed to confirm the activity of bone remodelling (Suvarnapathaki et al., 2022; Ding et al., 2021).

To evaluate *in vivo* angiogenesis within the calvarial samples, we performed immunofluorescence staining using specific markers, namely, endothelial cell adhesion molecule 1 (CD31) and alpha-smooth muscle actin (α-SMA). The tissue sections were incubated with primary antibodies against CD31 (1:500, GB11063-2, Servicebio) and α-SMA (1:100, GB12045, Servicebio), followed by counterstaining with DAPI (G1012, Servicebio) to visualise the cell nuclei. The images presented in this study were captured via an upright fluorescence microscope (Nikon Eclipse ci, Japan), and the mean fluorescence intensity was quantitatively analysed via ImageJ software. The early reconstitution of vascular networks is crucial for bone growth, and the formation of H-type vascular structures plays a key role in osteogenesis (Kusumbe et al., 2014; Zhang et al., 2020). Therefore, for the tissue sections obtained at week 4, immunofluorescence staining was performed using primary antibodies against CD31 and endomucin (EMCN, 1:100, GB112648, Servicebio). After image acquisition, the fluorescence intensity was analysed via ImageJ.

### 2.6 Statistical analysis

The experiments were performed in quadruplicate and repeated in quadruplicate unless indicated otherwise. The quantitative data are presented as the means ±standard deviations. Tests were analysed via GraphPad Prism software (Version 10, Boston, USA). Differences between two groups were assessed using Student’s t test, and those among multiple groups were analysed via one-way or two-way analysis of variance. Tukey’s HSD comparison test was used when multiple comparisons were performed. Differences were considered statistically significant at *P* < 0.05.

## 3 Results

### 3.1 *Micromorphology of* the scaffolds

**FIGURE 1:**
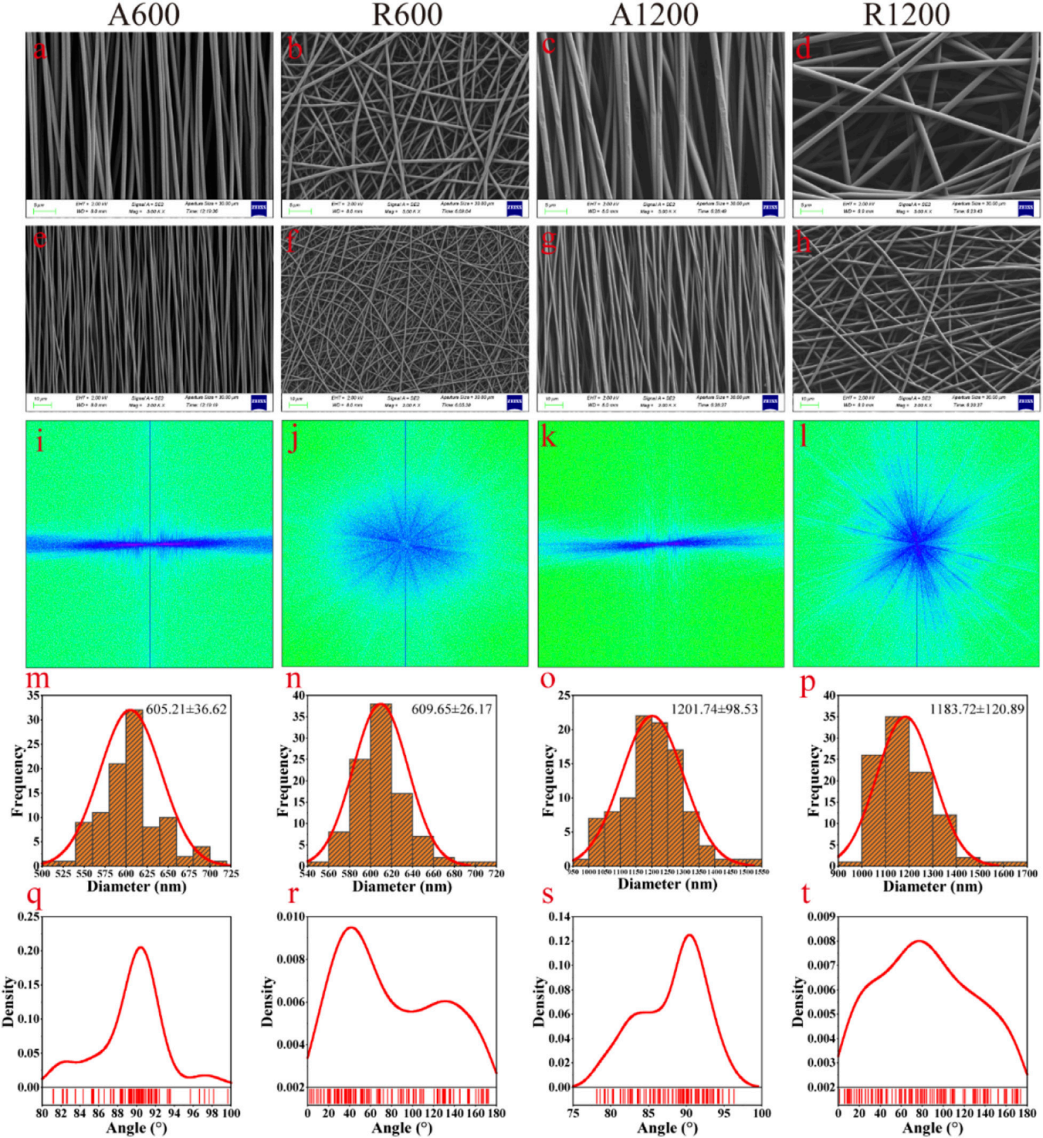
Characterisation of the scaffolds with different morphologies. **(A–H)** SEM images of scaffold membranes with different fibre diameters and orientations; scale bars, **(A–D)**: 5 μm, **(E–H)**: 10 μm. **(I–L)** 2D FFT output images. **(M–P)** Fibre diameter distributions of different scaffolds (n = 100). **(Q–T)** Fibre orientation distributions of different scaffolds (n = 100).

**FIGURE 2:**
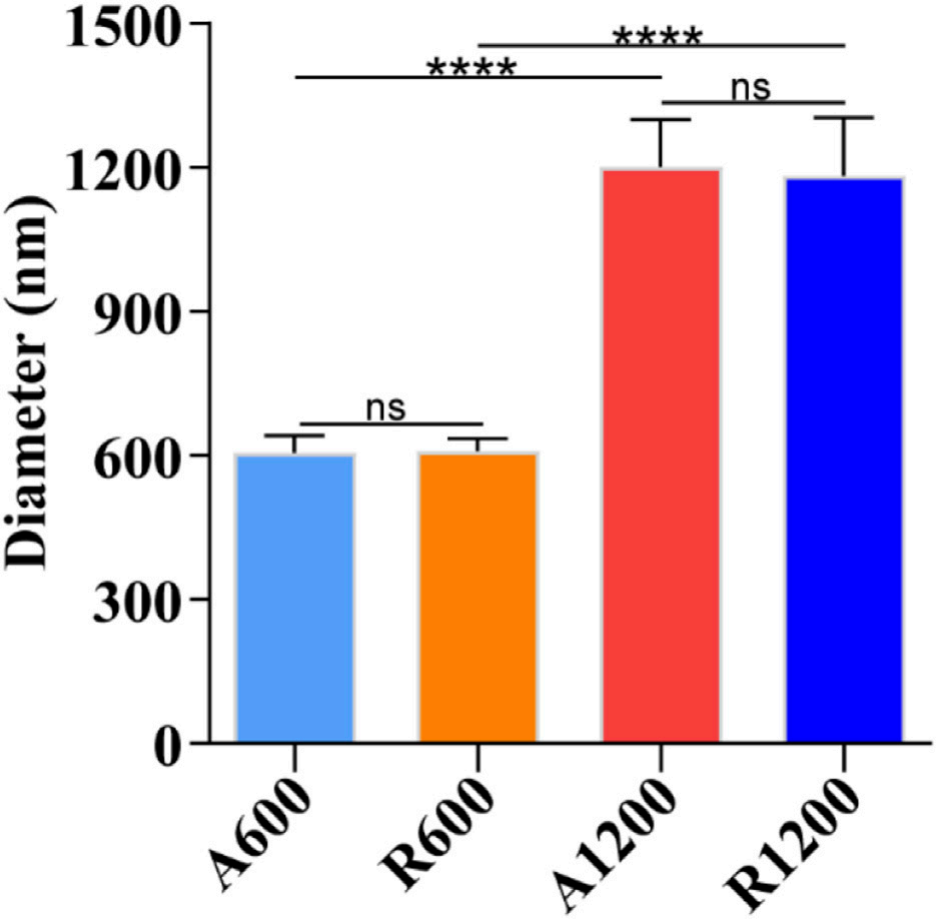
Fibre diameter analysis of different scaffolds (n = 100); ns represents no significant difference, ****P < 0.0001 represents the comparison between different scaffolds.

The SEM images of the scaffolds revealed a smooth and uniform surface. The A600 and A1200 scaffolds exhibited a concentrated orientation trend (Figures 1A, B, E, F), whereas the R600 and R1200 scaffolds exhibited a disordered arrangement characterised by random orientations (Figures 1C, D, G, H). Further analysis revealed that the distribution of fibre diameters across all the groups was close to a normal distribution (Figures 1M–P). Additionally, the fibre diameters for A600 and R600 were 605.21 ± 36.62 nm and 609.65 ± 26.17 nm, respectively, with no significant difference between the two groups. Similarly, the fibre diameters of A1200 and R1200 were 1201.74 ± 98.53 nm and 1183.72 ± 120.89 nm, respectively, with no significant difference between the two groups. Notably, a difference between the aligned and random groups was observed (*P* < 0.0001) (Figure 2). Furthermore, the angular distributions of A600 and A1200 were relatively concentrated, suggesting that a certain trend toward orientation concentration was achieved. However, the angular distributions of R600 and R1200 were relatively random (Figures 1Q–T). The characteristics of the highly concentrated orientation of A600 and A1200 were also confirmed by the 2D FFT results, which were considerably different from the random orientation characteristics of the R600 and R1200 scaffolds (Figures 1I–L).

### 3.2 *In vivo* evaluation of bone remodelling

**FIGURE 3:**
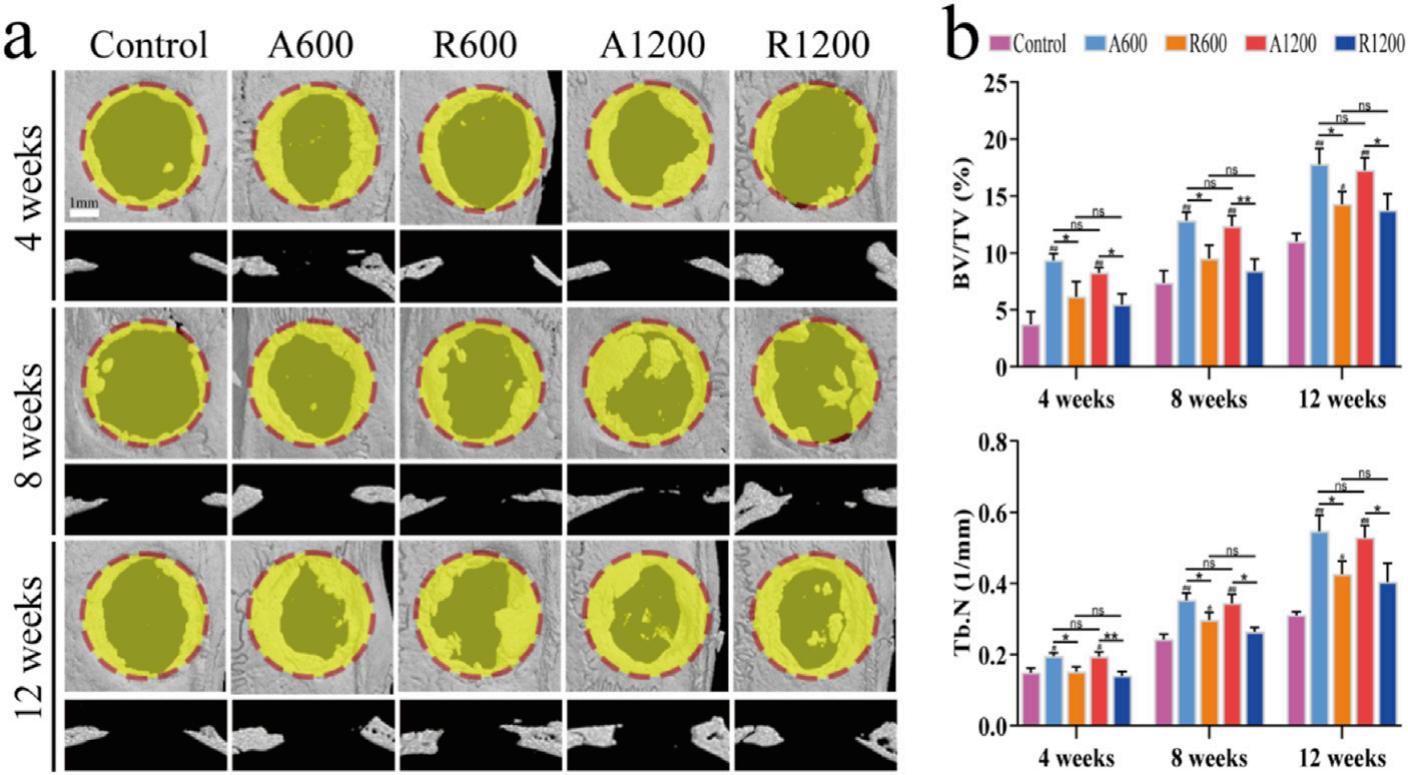
General evaluation of *in vivo* performance. **(A)** Micro-CT 3D reconstructed images at weeks 4, 8 and 12. **(B)** Quantification of BV/TV and Tb.N in the calvarial defect centres at weeks 4, 8 and 12; ns represents no significant difference; ^#^ *P* < 0.05, ^##^ *P* < 0.01 *versus* the control group; *P < 0.05, **P < 0.01 represents the comparison between different scaffolds.

Micro-CT scanning and 3D imaging reconstructions were performed on the calvarial specimens from each group of rats at specified time intervals. By week 4, new bone growth was observed at the edges of the defects in all the groups. Additionally, the aligned groups presented a significantly greater degree of growth than did the random groups. Nevertheless, the effect of the fibre diameter was only slightly evident when R600 was compared with R1200. The results from weeks 8 and 12 were largely consistent. Additionally, bone growth was greater in the aligned groups than in the random groups. Furthermore, the scaffold-implanted groups presented increased bone growth compared with the control group. Notably, no significant difference was observed in terms of fibre diameter (Figure 3A). The analysis of the bone volume fraction and trabecular number revealed that the aligned groups exhibited better bone growth than the control group at all time points, and the difference was statistically significant. There was a significant difference between the R600 group and the control group at week 12. Furthermore, at all the observed time points, a significant difference between the aligned and random groups was detected. However, for the groups with different diameters, the A600 and R600 groups presented higher values than the A1200 and R1200 groups, but a significant difference was not found (Figure 3B).

**FIGURE 4:**
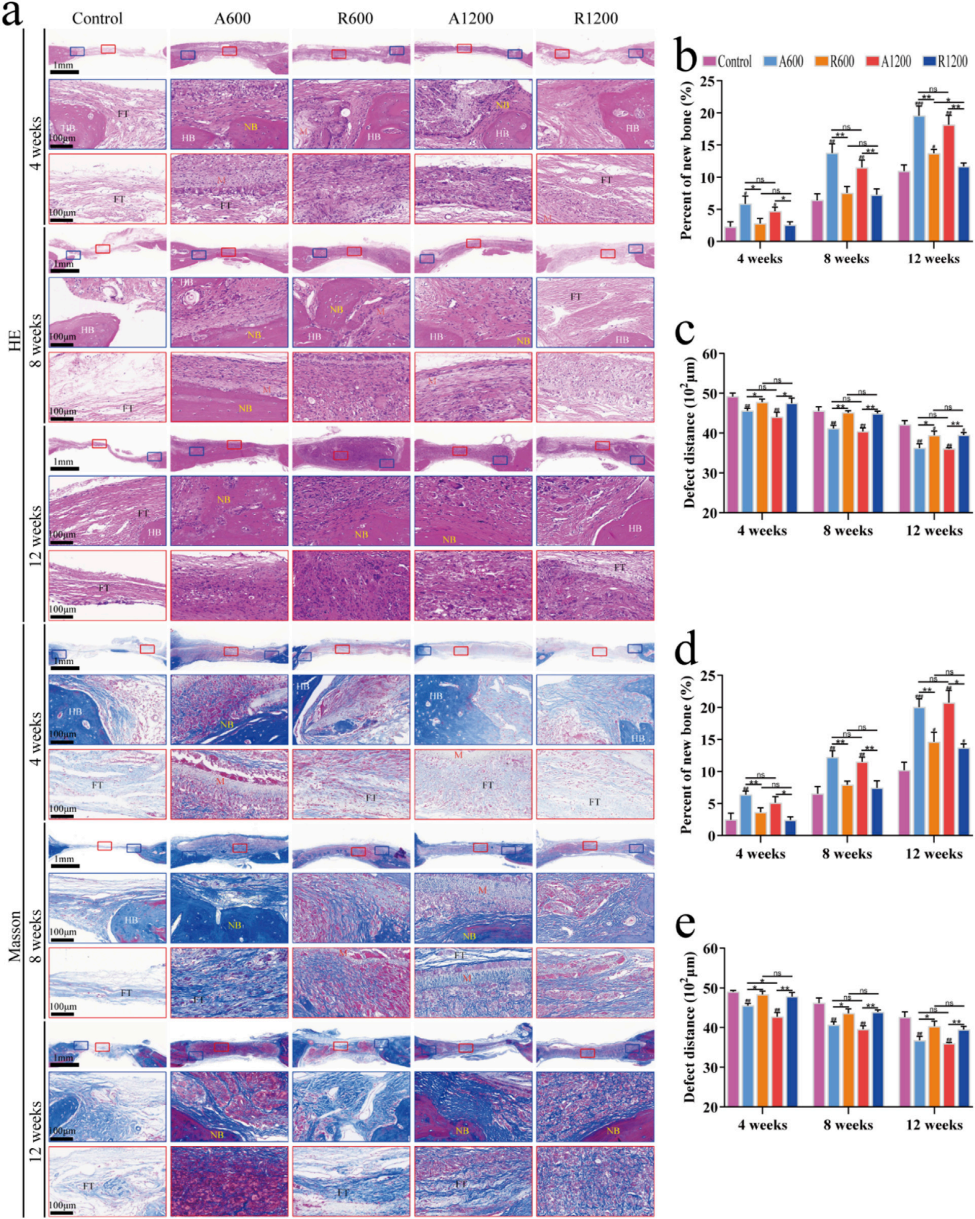
Histological analysis of bone remodelling *in vivo*. **(A)** Representative images showing H&E staining and Masson’s trichrome staining; HB indicates host bone, NB indicates new bone, M indicates the scaffold, and FT indicates fibrous tissue. **(B, C)** Quantification of the bone formation area and defect distance in the tissue samples stained with HE. **(D, E)** Quantification of the bone formation area and defect distance in the Masson-stained tissue samples; ns represents no significant difference; ^#^ *P* < 0.05, ^##^ *P* < 0.01, ^###^ *P* < 0.001 *versus* the control group; *P < 0.05, **P < 0.01 represents the comparison between different scaffolds.

**FIGURE 5:**
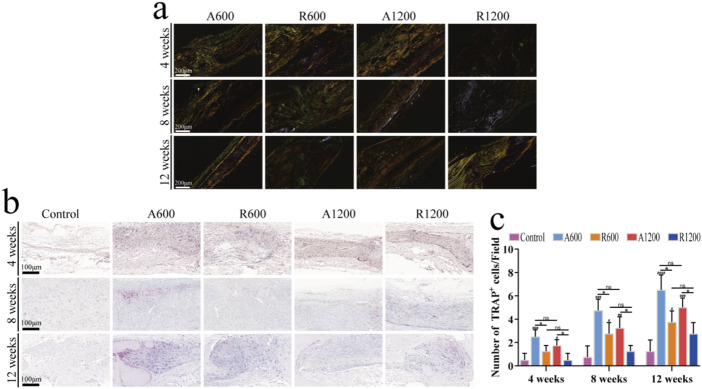
Observation of bone remodelling strength and collagen fibre growth *in vivo*. **(A)** Representative image of collagen fibre growth at weeks 4, 8 and 12. **(B)** Representative TRAP staining images of the calvarial defect centres at weeks 4, 8 and 12. **(C)** The number of TRAP^+^ cells in the defect regions; ns represents no significant difference, ^#^ *P* < 0.05, ^##^ *P* < 0.01, ^###^ *P* < 0.001 *versus* the control group, *P < 0.05 represents the comparison between different scaffolds.

H&E and Masson’s trichrome staining clearly revealed the morphology of new bone tissue in the bone defect regions, which is an effective method for evaluating bone regeneration. Further analysis via H&E and Masson staining revealed that the defect regions in the control group primarily consisted of fibrous connective tissues with minimal new bone formation at all time points. Nevertheless, the scaffold-implanted groups exhibited varying degrees of new bone formation. Gradually, the connection between the new bone and the original bone became pronounced. Moreover, because of the substantial deposition of the collagen matrix, Masson’s trichrome staining revealed a red colour at later time points (Wu et al., 2020) (Figure 4A). The results of the quantitative analysis of the new bone region and the defect length within the defect zone are consistent with the staining results (Figures 4B–E). All the groups gradually developed varying degrees of new bone formation, and the bone defect length progressively decreased. However, comparisons were made between the groups with different diameters, and significant differences were not found throughout the entire timeline. The aligned groups were significantly different from the control and random groups, whereas the random groups were significantly different only from the control group at week 12. This could be due to the polylactic acid scaffolds preventing the invasion of soft tissue, which creates conditions that are favourable for bone regeneration and affects osteogenic outcomes (Wu et al., 2020). TRAP staining revealed varying degrees of osteoclast activity throughout the process. Compared with that in the random groups, the osteogenic remodelling activity was greater in the aligned groups at all time points. Similarly, compared with the groups with large diameters, the groups with small diameters presented numerically greater values; however, the difference was not statistically significant (Figures 5B, C). Observations of the growth trend of the collagen fibres under polarised light microscopy revealed that in the aligned groups, the collagen fibres grew about along the longitudinal axis of the calvarial defect, whereas in the random groups, the collagen fibres grew along the pre-existing bone (Figure 5A). These findings support the theory that the aligned scaffold can affect collagen deposition throughout the bone regeneration process (Perikamana et al., 2015).

### 3.3 *In vivo* evaluation of angiogenesis

**FIGURE 6:**
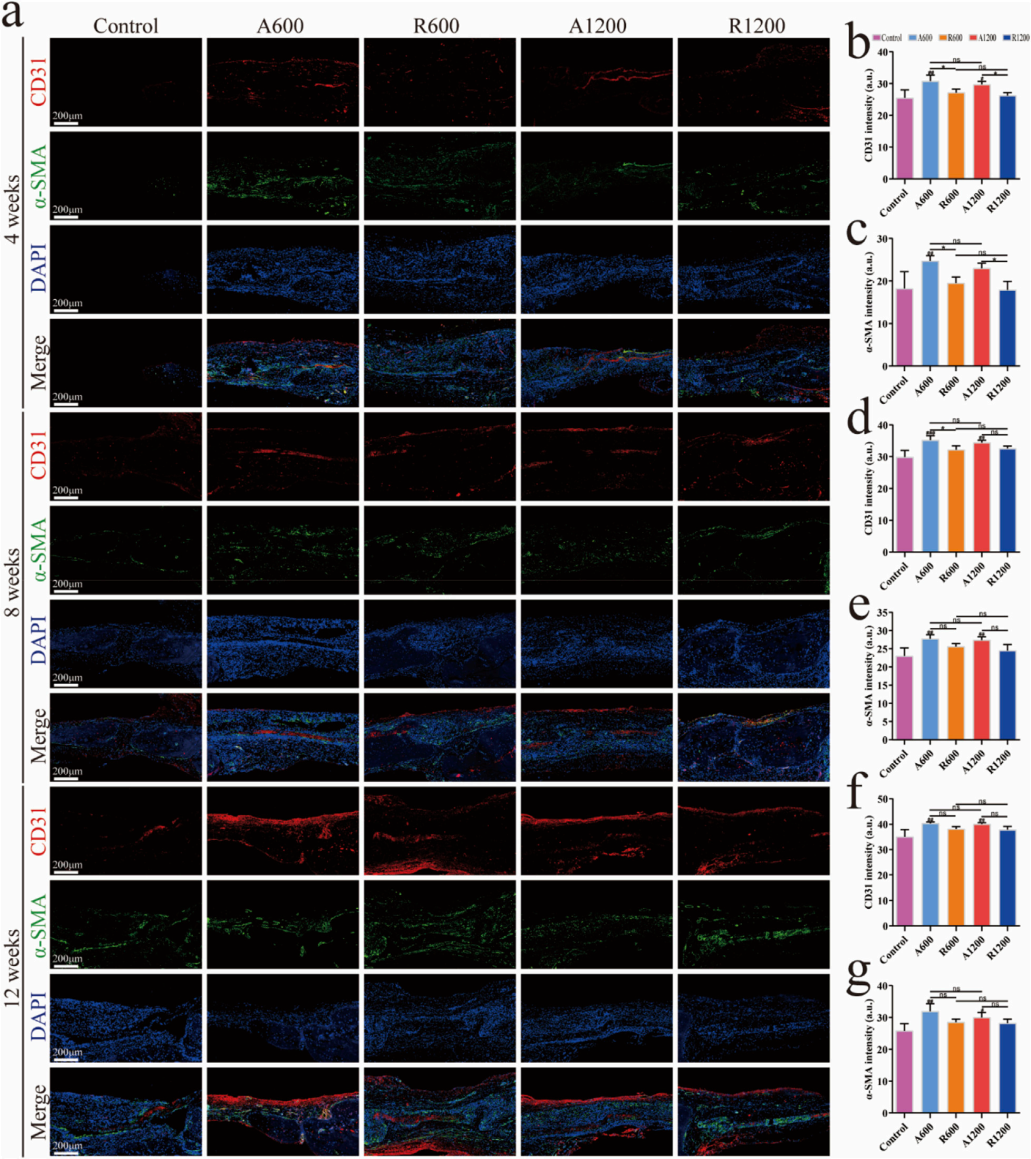
Evaluation of angiogenesis *in vivo*. **(A)** Immunofluorescence staining of CD31 (red), α-SMA (green) and DAPI (blue) at weeks 4, 8 and 12. **(B–G)** Quantitative analysis of CD31 and α-SMA at weeks 4, 8 and 12; ns represents no significant difference; ^#^ *P* < 0.05, ^##^ *P* < 0.01, ^###^ *P* < 0.001 *versus* the control group; *P < 0.01 represents the comparison between different scaffolds.

**FIGURE 7:**
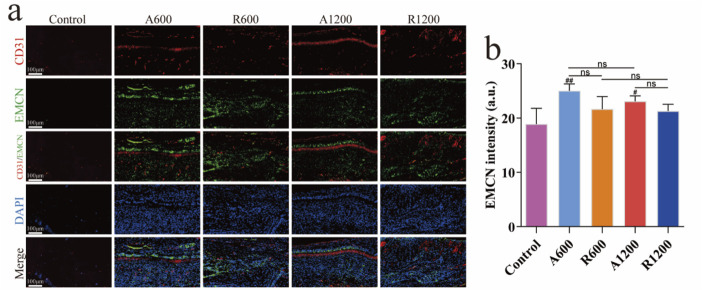
Evaluation of H-type vessels. **(A)** Immunofluorescence staining of CD31 (red), EMCN (green) and DAPI (blue) at week 4. **(B)** Quantitative analysis of EMCN at week 4; ns represents no significant difference, ^#^ *P* < 0.05, ^##^ *P* < 0.01 *versus* the control group.

The quantitative analysis of the immunofluorescence results revealed no significant difference among the groups of different diameters at each time point. By week 4, a significant difference was observed between the aligned and random groups, which gradually decreased. However, a significant difference was still found between the aligned and control groups (Figures 6A, B). EMCN analysis revealed a significant difference between the aligned group and the control group (Figures 7A, B), suggesting that the aligned scaffolds had a positive effect on angiogenesis during the early stages. This provides a background for superior osteogenic outcomes compared with those of the control and random groups in the later stages.

## 4 Discussion

In this study, we aimed to determine the effects of different micromorphologies of fibre scaffolds, including various orientations and diameters, on the process of bone regeneration. Bone is primarily composed of the inorganic component hydroxyapatite and the organic component type I collagen. During the process of bone regeneration, hydroxyapatite crystals are periodically deposited in the gap regions of collagen fibres, forming mineralised collagen fibre arrays in an oriented fibre array (Liu et al., 2016; Xie et al., 2021b). The oriented arrangement structure is considered a major factor in inducing the generation of anisotropic bone tissue and is highly important for the formation of the bone microstructure (Matsugaki et al., 2013). The application of aligned scaffolds has been extensively studied in the fields of tendon regeneration, periodontal repair, and neuronal differentiation (Ren et al., 2017; Chi et al., 2022). Furthermore, the effect of fibre diameter on osteogenic differentiation has also been widely investigated *in vitro* (Xie et al., 2021a; Chen et al., 2023; Gu et al., 2023).

In the present study, we prepared polylactic acid scaffold materials with various orientations and fibre diameters, and their influence on bone generation *in vivo* was systematically evaluated. We found that although the overall osteogenic efficacy was relatively low, the aligned fibre scaffolds exhibited superior bone repair effects to the random fibre scaffolds did, which supported the findings of previous studies. These findings suggest that aligned fibre scaffolds can guide the stimulation of tissue regeneration and provide spatial guidance for bone regeneration *in vivo* (Lee et al., 2014). Additionally, type I collagen fibres in natural bone, tendons, and ligaments show a consistent orderly arrangement spatially and directionally (Luo et al., 2021). Previous studies have shown that the random group and the aligned group have anisotropic characteristics in terms of Young’s modulus and contact hardness, which can direct the assembly and deposition of collagen (Perikamana et al., 2015; Zhang et al., 2019). Additionally, the orderly deposition of collagen fibres may act synergistically with the aligned scaffolds to guide cell migration in the collagen matrix direction (Lee et al., 2014), leading to better osteogenic effects in the groups with aligned scaffold implantation. Furthermore, comparative analysis between nanoscale and microscale fibre scaffolds cannot reveal a pronounced osteogenic effect. However, the groove dimension of a scaffold material can significantly affect bone regeneration *in vivo* (Lee et al., 2018). This finding is quite different from those of previous *in vitro* studies, suggesting that nanoscale fibre scaffolds can be more effective at promoting osteogenic differentiation than their microscale counterparts (Xie et al., 2021a). The possible reason could be that the enhancing effects of nanofibre diameters on cell attachment, proliferation, and differentiation are decreased within the complex *in vivo* environment.

The design of scaffold materials should closely follow the fundamental structure of natural bone tissue. If the material can provide microstructural factors conducive to the generation of new bone without the introduction of any other substances (Lee et al., 2018), it can further improve research on bone tissue engineering. The natural bone tissue is anisotropic (Lee et al., 2020), and orderly scaffolds with concentrated orderly orientation can exert a positive effect on the anisotropy of the newly formed bone tissue, suggesting that the intrinsic morphological structure of the material plays a major role in bone regeneration. The bone continuously undergoes remodelling and renewal via the coordination and cooperation between osteoblasts and osteoclasts (Peng et al., 2023), which is different from the improved osteoclastic activity identified as a potential starting point for bone regeneration in previous studies (Ding et al., 2021). The present study reports the complete experimental period through osteoclast activity, revealing ongoing bone remodelling activity in the bone defect area. However, the inherent absence of osteogenic activity in polylactic acid is a limitation of the present study. Therefore, the calvarial defect in the rat model persisted throughout the experimental period, and the bone remodelling consistently operated at a subdued level.

The endothelial cells in natural blood vessels exhibit a directional arrangement (Whited and Rylander, 2014). However, compared with *in vitro* studies on the effects of different orientations and diameters on endothelial cell fusion (Li et al., 2018), angiogenesis only differed between the aligned and random groups in the early stages of the experiment. Additionally, no significant effect of fibre diameter on angiogenesis was observed. We hypothesised that the effect of orientation on angiogenesis is derived mainly from the morphological modulation of the extracellular matrix or the secretory functions of mesenchymal stem cells *in vivo* (Lee et al., 2014); hence, angiogenesis can be affected. Additionally, angiogenesis is well known to follow the micro-paths or channels within the extracellular matrix environment (Czirok, 2013), and the aligned micromorphology may play a role to some extent. Furthermore, the recovery of blood perfusion in ischemic limbs of rats can be effectively increased by aligned fibre scaffolds loaded with endothelial cells (Nakayama et al., 2015), suggesting that scaffolds with a concentrated micromorphological orientation have a positive effect on vascular recovery. The most logical explanation for the less pronounced effect achieved in this study is the absence of exogenous cellular or bioactive components. Moreover, through the observation of H-type vessels, we found a certain synergistic effect between the aligned scaffolds and angiogenesis and osteogenesis.

In summary, in the present study, we successfully prepared polylactic acid fibre scaffolds with various orientations and diameters. The scaffolds with a concentrated orientation exhibited a positive effect on bone regeneration within the body and during early vascular formation, whereas comparative analysis between nanoscale and microscale scaffolds within the *in vivo* experiment revealed no significant differences in terms of vascular generation and bone regeneration, suggesting that materials with oriented structures have potential for future applications in bone defect repair. Additionally, the micromorphological structures of biomimetic scaffold materials can provide physical cues, valuable insights and novel strategies for the effective design and construction of materials in the future.

## Data Availability

The original contributions presented in the study are included in the article/supplementary material, further inquiries can be directed to the corresponding author.
